# The effects of an empathy role-playing program for operating room nursing students in Iran

**DOI:** 10.3352/jeehp.2018.15.29

**Published:** 2018-12-13

**Authors:** Negin Larti, Elaheh Ashouri, Akram Aarabi

**Affiliations:** 1 Department of Operating Room Nursing, School of Nursing and Midwifery, Isfahan University of Medical Sciences, Isfahan, Iran; 2 Department of Adult Health Nursing, Nursing and Midwifery Care Research Center, School of Nursing and Midwifery, Isfahan University of Medical Sciences, Isfahan, Iran; 3 Department of Operating Room Nursing, Nursing and Midwifery Care Research Center, School of Nursing and Midwifery, Isfahan University of Medical Sciences, Isfahan, Iran; Hallym University, Korea

**Keywords:** Empathy, Iran, Operating rooms, Role playing, Students

## Abstract

**Purpose:**

The purpose of this study was to investigate the effects of a role-playing training program conducted among operating room nursing students on empathetic communication with patients through measurements of empathy scores.

**Methods:**

This study was carried out among 77 operating room nursing students from the first to the fourth years studying at the School of Nursing and Midwifery of Isfahan University of Medical Sciences in the academic year 2017–2018. The intervention administered to the experimental group included a 12-hour training program on expressing empathy to patients that incorporated roleplaying. The Jefferson Scale of Empathy-Health Profession Student version was completed by the participants before, immediately after, and 1 month after the intervention. A comparative analysis of these 3 time points was conducted.

**Results:**

No significant difference was found in the total pre-intervention mean empathy scores before the intervention between the control group and the experimental group (P= 0.50). However, the total mean empathy scores in the experimental group immediately after and 1 month after the intervention were higher than those in the control group (P< 0.001).

**Conclusion:**

Empathy training through a role-playing technique was effective at improving the empathy scores of operating room nursing students, and this finding also underscores the fact that empathy can be promoted by education. Changing the educational curriculum of operating room nursing students is suggested in order to familiarize them with the concept of empathy in the operating room.

## Introduction

Empathy is assumed to be an essential component of the relationships between healthcare providers and patients [1], and it is thought to facilitate the development of mutual trust, increase patient satisfaction [2], and ultimately result in positive therapeutic outcomes for patients [3]. In this respect, studies have suggested that it might be possible to promote empathy through training courses if adequate educational opportunities are available [4]. Nonetheless, a meta-analysis conducted by researchers at the University of Michigan of results over the past 30 years found that today’s undergraduate students show less empathy than those of previous generations [5]. Some of the reasons for reduced empathy might include the lack of role models in the field, workload and time constraints, ignorance of the role of empathy in patient care, and the increasing ubiquity of technology-based therapies. Furthermore, researchers believe that technology-based treatments are likely to affect the degree to which providers pay attention to the human dimension of patient communication during therapeutic processes [6]. In this regard, the operating room is an environment in the hospital where technology-centeredness is especially evident. Working in the operating room is different from working in other wards. The caring role of surgical technologists as healthcare providers in the operating room has been significantly influenced by technology [7]. A study by Gilmartin and Wright [8] showed that most patients sometimes felt abandoned in the preoperative stage, and that healthcare providers had failed to provide them with psychological support in this stage. Entering the operating room is often associated with fear and anxiety for many patients, and despite the limited time that surgical technologists have to interact with patients before the induction of anesthesia, it should be possible for them to communicate within this short period of time in a way that helps calm down anxious patients [9]. In a patient care program released by the Association of Perioperative Registered Nurses, the creation of a caring atmosphere by showing empathy to patients was introduced as a useful factor for reducing the anxiety levels of patients in the operating room prior to surgery [9]. Santos et al. [10] also suggested that healthcare providers in the operating room should cultivate empathy as part of providing care for patients. Therefore, showing empathy to patients in the operating room seems to have an effect on healthcare provider-patient communications, and teaching this concept to operating room nursing students as would-be personnel in this domain is of the utmost importance.

Various techniques have been proposed for empathy training, such as improving interpersonal skills, audio- or video-taping of patient encounters, interacting with role models, experiencing hospitalization, studying literature and the arts, engaging in theatrical performances, and role-playing [3]. In this respect, role-playing is used in teaching as a type of simulation that helps learners to experience and practice the process of making decisions regarding difficult situations in a stress-free environment [11]. Role-playing scenarios can similarly provide a safe environment for teaching care and empathy concepts to students in order to engage them actively in the learning process [12]. Although studies have investigated empathy among students in multiple medical disciplines [4,13], to our knowledge, no study has been conducted on empathy training for operating room nursing students. Therefore, the purpose of this study was to investigate the effects of a role-playing training program for empathetic communication with patients on the empathy scores of operating room nursing students.

## Methods

### Ethical statement

This study was approved by the Ethics Committee of Isfahan University of Medical Sciences, Isfahan, Iran (IR.MUI.REC.1396.3.839). The study was also registered with the Iranian Registry of Clinical Trials (IRCT20180519039726N1). We explained the objectives to the participants and received their written consent for participation in the study. The participants were further assured that their information would be kept confidential.

### Study design

A comparative study was carried out between 2 groups with a pretest-posttest design and 1-month follow-up after the intervention in the academic year 2017–2018 among 77 operating room nursing students from the first to the fourth years studying at the School of Nursing and Midwifery of Isfahan University of Medical Sciences, Isfahan, Iran.

The present study was not conducted as part of a university course for students; instead, it was held as a separate extracurricular training program. Accordingly, participation in all stages of the research was completely voluntary, and the students were assured that their presence or absence, as well as their scores on the questionnaires, would not have any impact on their academic evaluations.

The study population in this research included 130 operating room nursing students in total. Considering a 95% confidence coefficient and 80% test power, the minimum sample size was 64 individuals (32 in each group). However, as the study required participation for 3 consecutive weeks and the researchers were concerned about the possibility of extensive sample loss, a probable loss of 28% was estimated and 82 students (41 in each group) were recruited.

Ultimately, 5 students in the experimental group did not complete the intervention and were excluded (2 due to minor sickness, 2 due to conflicting class schedules, and 1 due to personal travel). Therefore, 36 students in the experimental group and 41 individuals in the control group (77 students in total) constituted the final study sample.

### Materials and/or subjects

Stratified random sampling was used. In order to perform the sampling, the names of operating room nursing students were obtained from the head of the Education Department, and students who met the inclusion criteria of the study (i.e., second-semester or higher students who had entered the stage of clinical practice, had experience with communicating with patients, had not been diagnosed with any psychological conditions, and had no history of participation in communication or patient empathy workshops) were selected. A number was then randomly assigned to each of the students, and the numbers were poured into a bowl. The first paper drawn out of the bowl was for the experimental group, the second paper was for the control group, and this procedure was continued to select students from all years of study. The exclusion criteria included incomplete responses to questionnaires, absence at any of the training sessions, and withdrawal from continuation of the study.

The research instrument was a 2-part questionnaire. The first section included demographic characteristics (age, gender, marital status, and years of study), and the second section contained the items of the Jefferson Scale of Empathy (JSE). The JSE has several versions for medical students, health professionals, health profession students, and nursing students. The different versions are similar in content, with slight changes in their wording [6].

Accordingly, the JSE-Health Profession Students version (JSE-HPS) was used in this study. The questionnaire was composed of 20 items distributed among 3 sub-scales: perspective-taking (10 items), compassionate care (8 items), and standing in patient’s shoes (2 items). This questionnaire also had 10 negative items that could be reversescored. Each item was scored on a 7-point Likert-type scale (from 1= strongly disagree to 7= strongly agree). The score for the entire questionnaire ranged from 20 to 140, with higher scores indicating more empathy with patients [6]. The validity and reliability of different versions of this questionnaire have been similarly confirmed in numerous investigations. According to Fields et al. [14], the Cronbach alpha coefficient of this scale was 0.78 and the test-retest reliability coefficient was 0.58 and 0.69 for 3-month and 6-month intervals, respectively. In the study by Shariat and Habibi [15], the Cronbach alpha coefficient and the test-retest reliability coefficient were 0.79 and 0.95, respectively, and confirmatory factor analysis upheld the original 3-factor structure (compassionate care, perspective-taking, and standing in patient’s shoes) consisting of 20 items. In the present study, the validity of the Persian version of the questionnaire was confirmed by 10 faculty members and its reliability was calculated using the Cronbach alpha coefficient, which was equal to 0.71.

### Technical information

The intervention for the experimental group included a training program for empathetic communication with patients in the operating room, mainly during the preoperative phase, using a role-playing technique. There were 3 training sessions lasting 4 hours each (12 hours in total) over the course of 3 consecutive weeks.

The researcher participated in a workshop entitled ‘Empathy’ which was held by a team of psychologists before the intervention and received a certificate. Furthermore, a psychologist specializing in holding empathy workshops assisted the researcher at all intervention sessions.

The content of the training sessions for the experimental group was selected in consultation with professors and researchers, as well as reviews of related studies [3,12], as follows:

*Session 1*: The participants first completed the JSE-HPS (as a pretest). The objectives of the training program were then explained. The topics included definitions of empathy (and empathy with patients), differences between empathy and sympathy, benefits of empathy with patients, empathy stages, situations requiring empathy, and the results of empathetic behaviors towards patients. Moreover, during the presentation of the topics, examples of empathy with patients in the operating room and situations wherein patients in the operating room needed empathy were mentioned and the study participants discussed them with each other. At the end of each session, the participants were encouraged to empathize with patients in their interactions until the next session, and the time and the place of the next session was specified.

*Session 2*: At the beginning of this session, the participants were asked about the uses of the topics discussed in the previous session. Then, the topics of the second session were explained, including techniques for showing empathy to patients and obstacles. These techniques included paying attention to patients’ faces, listening to patients, receiving reflections and feelings from patients, not judging patients, and paying attention to patients’ suffering, but not their behaviors. To review the techniques presented in this session, the students discussed and exchanged their ideas concerning a hypothetical or a real issue in their lives for which they needed empathy in groups of 2 in order to consolidate the techniques in their minds and to prepare themselves for the next session, in which they were required to empathize using role-playing.

Since the goal was to learn techniques related to empathy, the purpose of the first and second sessions was to present theoretical issues relating to empathy, along with a discussion of methods, group work, and question and answer sessions in order to prepare the participants for role-playing in the third session.

*Session 3*: The classroom was prepared by the researcher to implement the role-playing scenarios. At the beginning of the session, the role-playing technique was explained to the participants, and the objectives of the session were also delineated. Within this session, which was held to practice the skills that they had learned to empathize with patients in the operating room, the 36 students in the experimental group were randomly assigned to 12 groups of 3 individuals, and they played roles based on 12 scenarios, pre-designed by the primary researcher, whose validity had been already approved by 10 faculty members. An example of these scenarios is illustrated in Table 1. First, each scenario was randomly selected by each group and it was practiced with group members, with 1 person playing the role of the patient and 2 individuals playing the role of surgical technologists in the operating room. Then, all the scenarios were implemented under the researchers’ guidance, and the roles were exchanged among the individuals in each group. Following the end of the presentations by each group, the role-playing scenario was discussed. At the end of this session, the participants responded to the JSE-HPS (post-test 1) once more. One month after the last session of the intervention, post-test 2 was administered to assess the durability of the effects of the training intervention in the experimental group.

The control group did not receive any interventions, and the participants merely responded to the JSE-HPS at 3 time points. In order to avoid the possibility that the training provided to the experimental group could affect the empathy scores of the control group, testing of the control group was conducted before the intervention had begun for the experimental group, and during this period, the experimental group was not informed about the details of the intervention.

For ethical reasons, the same training session was offered to the control group if they desired, and the training session was made available after the experimental group completed post-test 2. Therefore, it was optional and not a part of this study.

### Statistics

Data analysis was performed via descriptive statistics (mean and frequency) and statistical tests (independent t-test, chi-square test, Mann-Whitney U-test, and the Bonferroni correction test) using IBM SPSS ver. 22.0 (IBM Corp., Armonk, NY, USA). P-values < 0.05 were considered to indicate statistical significance.

## Results

The demographic characteristics of the 77 study participants are presented in Table 2. Based on the results of the independent t-test, the Mann-Whitney U-test, and the chi-square test, the 2 groups were non-significantly different in terms of age (P= 0.24, t= 1.17), years of study (P= 0.88, Z= 0.15), sex (P= 0.70, χ^2^= 0.15), and marital status (P= 0.36, χ^2^= 0.84) (Table 2).

According to the results of the independent t-test, the total mean empathy scores and its dimensions before the intervention did not demonstrate significant differences between the control and the experimental groups (P> 0.05); however, the total mean empathy score and its dimensions in the experimental group were significantly higher than in the control group immediately after and at 1 month after the intervention (P< 0.05) (Table 3).

Moreover, a comparison of the total mean empathy scores in the experimental group between paired time intervals using the Bonferroni correction test showed that the total mean empathy score and its dimensions immediately after and at 1 month after the intervention were significantly higher than the corresponding scores before the intervention (P< 0.05); however, there was no significant difference between the mean scores immediately after and at 1 month after the intervention (P> 0.05) (Table 4). The raw data are available in Supplement 1.

## Discussion

This study was conducted to determine the effect of an empathy role-playing program on empathy scores among operating room nursing students. The total mean empathy score in the experimental group significantly increased immediately after the intervention compared to the control group. In line with the present study, in a study that aimed to investigate the effect of participation in an aging simulation game on nursing students’ empathy, mean empathy scores toward older adults assessed using the JSE-HPS significantly improved after the intervention [16]. Furthermore, Singh et al. [17] investigated the effect of using role-playing scenarios as an empathy training tool for ophthalmology postgraduates in a study where 39 individuals received an educational intervention during 2 sessions (4 hours) about empathy and observed 5 pre-designed scenarios played by trained students. The scenarios dealt with common ophthalmology cases that required counseling or care. The JSE questionnaire was completed by the participants before and 6 weeks after the intervention. The total mean empathy score was significantly higher at 6 weeks after the intervention (106.7± 17.5) than before the intervention (95.9±18.4) (P< 0.0001) [17]. Empathy is defined as a predominantly cognitive feature that includes understanding patients’ experiences and concerns, along with the ability to convey this understanding [3]. Since understanding and cognition can be influenced by education, empathy is also considered to be a skill that can be trained [6].

In addition to the total mean empathy score in this study, the scores for all its dimensions (perspective-taking, compassionate care, and standing in patient’s shoes) after the intervention in the experimental group were significantly higher than those in the control group. This may have occurred because the intervention required students to play the role of the patient, thus helping them realize patients’ needs in certain situations; therefore, the scores of the sub-scales in which the experience of receiving care could be reinforced via placing oneself in the role of a patient significantly increased. Chen et al. [16] suggested that “students may not be aware of older adults’ feelings prior to experiencing aging-related changes themselves, and simulation activities can be a useful method to allow students to ‘walk in the shoes’ of an older patient.” However, we did not find any study that investigated empathy sub-scales after an educational intervention in a way that would be directly comparable with our results.

The improved empathy scores among the participants of this study were not merely the result of theoretical training about empathy or the effects of the role-playing technique by itself. Instead, teaching the concept of empathy to operating room nursing students, who had previously received no education about empathy, requires learning techniques and theoretical issues and then consolidating skills through practice (using the role-playing technique in this study). Therefore, integrating theoretical issues with the role-playing technique could affect empathy scores in participants after the intervention. Since the operating room can be a stressful learning environment for students, the use of engaging training techniques, such as role-playing, can be helpful for students.

Regarding the durability of the empathy scores, the results of the present study indicated that the total mean empathy score and its dimensions at 1 month after the intervention were significantly higher in the experimental group than in the control group. Additionally, the total mean empathy score in the experimental group at 1 month after the intervention (122.11) showed an improvement compared to the scores before (107.31) and immediately after the intervention (120.58). The durability of the empathy scores in the present study could be explained as resulting from the fact that the operating room nursing students had not received academic education on empathy with patients during their studies; therefore, they became motivated and interested in this subject after the intervention. Moreover, their empathy scores increased as they entered clinical settings, encountered patients, and practiced these skills. Although the increase in the empathy scores of participants on posttest 2 compared to posttest 1 could have been affected by their entry into the clinical setting, their initial familiarity with this concept may have been shaped by this intervention. This possibility should be considered because an objective of this study was to foster sensitivity about this concept among operating room nursing students in order to encourage them to communicate empathetically in clinical settings and when communicating with patients. In accordance with the present study, Bas-Sarmiento et al. [1] performed a follow-up study on participants’ empathy scores 1 month after a 20-hour empathy training program, and found that the empathy scores in the follow-up phase were significantly higher than those before the intervention. Contrary to the findings in the present study, van Winkle et al. [13] found that although the empathy scores of participants immediately after the implementation of a 40-minute workshop on empathy using the role-playing technique were significantly higher than those before the intervention, this increasing trend was not persistent; in the second stage of the posttest it returned to the pretest level, and a significant change was not found. A possible explanation for the instability of those scores might be the short duration of training in the study by van Winkle et al. [13] (40 minutes), compared to the training programs in the present study (12 hours) and the investigation by Bas-Sarmiento et al. [1] (20 hours).

In this study, the total mean empathy score among operating room nursing students before the intervention was 107.31 in the experimental group and 105.95 in the control group. Although no comparable study specifically aiming at assessing empathy in operating room nursing students was found, these scores were in agreement with those reported by Williams et al. [2] in a study of undergraduate paramedic students (108.60) using the JSE-HPS, McKenna et al. [18] in a study of nursing students (107.34) with the Jefferson Scale of Physician Empathy, Health Professional version, and by Hegazi and Wilson [19] in a study of medical students (109.07) using the Jefferson Scale of Physician Empathy, Student version.

In conclusion, the present study showed that the implementation of a training program using a role-playing technique could be effective for improving empathy scores among operating room nursing students. It is therefore suggested that changes should be made in the curriculum of operating room nursing students in order to familiarize them with the concept of empathy and to encourage them to pay attention to the human dimension of patient care in the operating room. Furthermore, it is proposed that the role-playing technique should be used as a teaching method for training operating room nursing students in clinical skills, since it is thought to be a useful, engaging, and cost-effective technique.

This study had some limitations. The participants could have encountered a movie or a story between the training sessions that affected their empathy scores, and such exposures were beyond our control. Since the participants were selected from all years of study and absence at even 1 session was an exclusion criterion, it was difficult for us to coordinate the classes in a way that students could be present at all sessions, impeding our ability to increase the number of participants or sessions.

## Figures and Tables

**Table 1.** A sample scenario of empathy with a patient in the operating room

A young man is transferred to the operating room to undergo surgical amputation following a car accident.
Patient: That day, at noon, I took my father’s motorcycle for a ride without his permission.
I was on a one-way street, and I didn’t realize it.
I did not see that a car was about to hit the motorcycle and I fell off.
They took me to the hospital.
My right leg was damaged and I underwent several operations.
Today, the doctors concluded that it needed to be amputated!!!

**Table 2.** Demographic characteristics of the experimental and control groups

Variable	Control (n = 41)	Experimental (n = 36)	P-value
Sex			0.70
Female	29 (70.7)	24 (66.7)	
Male	12 (29.3)	12 (33.3)	
Marital status			0.36
Single	32 (78.0)	31 (86.1)	
Married	9 (22.0)	5 (13.9)	
Years of study			0.88
First	10 (24.4)	10 (27.8)	
Second	11 (26.8)	8 (22.2)	
Third	10 (24.4)	7 (19.4)	
Fourth	10 (24.4)	11 (30.6)	
Age (yr)	20.83 ± 1.51	21.44 ± 2.95	0.24

Values are presented as frequency (%) or mean±standard deviation.

**Table 3.** Comparison of the total mean empathy score and its dimensions before, immediately after, and at 1 month after the intervention between the experimental and control groups

Dimension	Before intervention				Immediately after intervention				One month after intervention			
	Control	Experimental	t-value	P-value	Control	Experimental	t-value	P-value	Control	Experimental	t-value	P-value
Total mean empathy score	105.95 ± 8.59	107.31 ± 8.97	0.68	0.50	103.98 ± 10.48	120.58 ± 12.47	6.35	< 0.001	105.02 ± 10.46	122.11 ± 12.05	6.66	< 0.001
Perspective-taking	53.83 ± 5.41	54.89 ± 5.38	0.86	0.39	52.37 ± 6.33	61.78 ± 5.79	6.77	< 0.001	53.61 ± 6.35	62.53 ± 6.53	6.07	< 0.001
Compassionate care	44.10 ± 4.79	43.89 ± 4.59	0.19	0.85	44.12 ± 4.89	48.25 ± 6.41	3.20	0.002	43.71 ± 4.88	48.53 ± 5.61	4.03	< 0.001
Standing in patient’s shoes	8.02 ± 1.99	8.53 ± 2.30	1.03	0.31	7.49 ± 2.57	10.56 ± 2.37	5.42	< 0.001	7.71 ± 2.21	11.06 ± 2.32	6.49	< 0.001

Values are presented as mean±standard deviation, unless otherwise stated.

**Table 4.** Comparison of the total mean empathy score and its dimensions in the experimental group between paired time intervals

Dimension	Paired time intervals	P-value
Total mean empathy score	Before and immediately after the intervention	< 0.001
	Before and at 1 month after the intervention	< 0.001
	Immediately after and 1 month after the intervention	0.94
Perspective-taking	Before and immediately after the intervention	< 0.001
	Before and 1 month after the intervention	< 0.001
	Immediately after and 1 month after the intervention	0.98
Compassionate care	Before and immediately after the intervention	< 0.001
	Before and 1 month after the intervention	< 0.001
	Immediately after and 1 month after the intervention	0.97
Standing in patient’s shoes	Before and immediately after the intervention	< 0.001
	Before and 1 month after the intervention	< 0.001
	Immediately after and 1 month after the intervention	0.35

## Supplementary materials

Supplement 1. Data files are available from https://doi.org/10.7910/DVN/LG8UPQ

Supplement 2. Audio recording of the abstract.
